# White Blood Cell Segmentation by Circle Detection Using Electromagnetism-Like Optimization

**DOI:** 10.1155/2013/395071

**Published:** 2013-02-13

**Authors:** Erik Cuevas, Diego Oliva, Margarita Díaz, Daniel Zaldivar, Marco Pérez-Cisneros, Gonzalo Pajares

**Affiliations:** ^1^Departamento de Electrónica, Universidad de Guadalajara, CUCEI, Avenida Revolución 1500, 44430 Guadalajara, JAL, Mexico; ^2^Departamento de Ingeniería del Software e Inteligencia Artificial, Facultad Informática, Universidad Complutense, Avenida Complutense S/N, 28040 Madrid, Spain

## Abstract

Medical imaging is a relevant field of application of image processing algorithms. In particular, the analysis of white blood cell (WBC) images has engaged researchers from fields of medicine and computer vision alike. Since WBCs can be approximated by a quasicircular form, a circular detector algorithm may be successfully applied. This paper presents an algorithm for the automatic detection of white blood cells embedded into complicated and cluttered smear images that considers the complete process as a circle detection problem. The approach is based on a nature-inspired technique called the electromagnetism-like optimization (EMO) algorithm which is a heuristic method that follows electromagnetism principles for solving complex optimization problems. The proposed approach uses an objective function which measures the resemblance of a candidate circle to an actual WBC. Guided by the values of such objective function, the set of encoded candidate circles are evolved by using EMO, so that they can fit into the actual blood cells contained in the edge map of the image. Experimental results from blood cell images with a varying range of complexity are included to validate the efficiency of the proposed technique regarding detection, robustness, and stability.

## 1. Introduction

Nature-inspired computing is a field of research that is concerned with both the use of biology as an inspiration for solving computational problems and the use of the natural physical phenomena to solve real world problems. Moreover, nature-inspired computing has proved to be useful in several application areas [1] with relevant contributions to optimization, pattern recognition, shape detection, and machine learning. In particular, it has gained considerable research interest from the computer vision community as nature-based algorithms have successfully contributed to solve challenging computer vision problems.

On the other hand, white blood cells (WBCs) also known as leukocytes play a significant role in the diagnosis of different diseases. Although digital image processing techniques have successfully contributed to generate new methods for cell analysis, which, in turn, have lead into more accurate and reliable systems for disease diagnosis, however, high variability on cell shape, size, edge, and localization complicates the data extraction process. Moreover, the contrast between cell boundaries and the image's background may vary due to unstable lighting conditions during the capturing process.

Many works have been conducted in the area of blood cell detection. In [2], a method based on boundary support vectors is proposed to identify WBC. In such approach, the intensity of each pixel is used to construct feature vectors whereas a support vector machine (SVM) is used for classification and segmentation. By using a different approach, in [3], Wu et al. developed an iterative Otsu method based on the circular histogram for leukocyte segmentation. According to such technique, the smear images are processed in the Hue-Saturation-Intensity (HSI) space by considering that the Hue component contains most of the WBC information. One of the latest advances in white blood cell detection research is the algorithm proposed by Wang et al. [4] which is based on the fuzzy cellular neural network (FCNN). Although such method has proved successful in detecting only one leukocyte in the image, it has not been tested over images containing several white cells. Moreover, its performance commonly decays when the iteration number is not properly defined, yielding a challenging problem itself with no clear clues on how to make the best choice.

Since blood cells can be approximated with a quasi-circular form, a circular detector algorithm may be handy. The problem of detecting circular features holds paramount importance for image analysis, in particular for medical image analysis [5]. The circle detection in digital images is commonly performed by the circular Hough transform [6]. A typical Hough-based approach employs an edge detector whose information guides the inference for circle locations and radius values. Peak detection is then performed by averaging, filtering, and histogramming the transform space. However, such approach requires a large storage space given by the required 3D cells to cover all parameters (*x*, *y*, *r*). It also implies a high computational complexity yielding a low processing speed. The accuracy of the extracted parameters for the detected circle is poor, particularly in presence of noise [7]. For a digital image holding a significant width and height and a densely populated edge pixel map, the required processing time for circular Hough transform makes it prohibitive to be deployed in real time applications. In order to overcome such a problem, some other researchers have proposed new approaches based on the Hough transform, for instance the probabilistic Hough transform [8, 9], the randomized Hough transform (RHT) [10] and the fuzzy Hough transform [11]. Alternative transformations have also been presented in the literature as the one proposed by Becker et al. in [12]. Although those new approaches demonstrated better processing speeds in comparison to the original Hough transform, they are still very sensitive to noise.

As an alternative to Hough transform-based techniques, the circle detection problem has also been handled through optimization methods. In general, they have demonstrated to deliver better results than those based on HT considering accuracy, speed, and robustness [13]. Such approaches have produced several robust circle detectors using different optimization algorithms such as genetic algorithms (GAs) [13], harmony search (HS) [14], differential evolution (DE) [15], and the electromagnetism-like optimization algorithm (EMO) [16]. 

Although detection algorithms based on the optimization approaches present several advantages in comparison to those based on the Hough transform, they have been scarcely applied to WBC detection. One exception is the work presented by Karkavitsas and Rangoussi [17] that solves the WBC detection problem through the use of GA. However, since the evaluation function, which assesses the quality of each solution, considers the number of pixels contained inside of a circle with fixed radius, the method is prone to produce misdetections particularly for images that contained overlapped or irregular WBC.

In this paper, the WBC detection task is approached as an optimization problem, and the EMO-based circle detector [16] is used to build the circular approximation. The EMO algorithm [18] is a stochastic evolutionary computation technique based on the electromagnetism theory. It considers each solution to be a charged particle. The charge of each particle is determined by an objective function. Thereby, EMO moves each particle according to its charge within an attraction or repulsion field among the population using Coulomb's law and the superposition principle. This attraction-repulsion mechanism of the EMO algorithm corresponds to the reproduction, crossover, and mutation in GA [19]. In general, the EMO algorithm can be considered as a fast and robust algorithm representing an alternative to solve complex, nonlinear, nondifferentiable and nonconvex optimization problems. The principal advantages of the EMO algorithm lie on several facts: it has no gradient operation, it can be used directly on a decimal system, it needs only few particles to converge, and the convergence existence has been already verified [20].

The EMO-based circle detector uses the encoding of three edge points that represent candidate circles in the edge map of the scene. The quality of each individual is calculated by using an objective function which evaluates if such candidate circles are really present in the edge map of the image. The better a candidate circle approximates the actual edge circle, the more the objective function value decreases. Therefore, the detection performance depends on the quality of the edge map as it is obtained from the original images. However, since smear images present different imaging conditions and staining intensities, they produce edge maps partially damaged by noisy pixels. Under such conditions, the use of the EMO-based circle detector cannot be directly applied to WBC detection.

This paper presents an algorithm for the automatic detection of blood cell images based on the EMO algorithm. The proposed method modifies the EMO-based circle detector by incorporating a new objective function. Such function allows to accurately measure the resemblance of a candidate circle with an actual WBC on the image which is based on the information not only from the edge map but also from the segmentation results. Guided by the values of the new objective function, the set of encoded candidate circles are evolved using the EMO algorithm so that they can fit into actual WBC on the image. The approach generates a subpixel detector which can effectively identify leukocytes in real images. Experimental evidence shows the effectiveness of such method in detecting leukocytes despite complex conditions. Comparison to the state-of-the-art WBC detectors on multiple images demonstrates a better performance of the proposed method.

The main contribution of this study is the proposal of a new WBC detector algorithm that efficiently recognizes WBC under different complex conditions while considering the whole process as a circle detection problem. Although circle detectors based on optimization present several interesting properties, to the best of our knowledge, they have not yet been applied to any medical image processing up to date.

This paper is organized as follows.  Section 2 provides a description of the EMO algorithm while in Section 3 the circle detection task is fully explained from an optimization perspective within the context of the EMO approach. The complete WBC detector is presented in Section 4.  Section 5 reports the obtained experimental results whereas Section 6 conducts a comparison between the state-of-the-art WBC detectors and the proposed approach. Finally, in Section 7, some conclusions are drawn.

## 2. Electromagnetism-Like Optimization Algorithm (EMO)

Initially designed for bound constrained optimization problems, the EMO method [18] utilizes *N*, *n*-dimensional points *x*_*i*,*k*_, *i* = 1, 2,…, *N*, as a population for searching the feasible set *X* = {*x* ∈ *R* ^*n*^ | *l*_*i*_ ≤ *x* ≤ *u*_*i*_}, where the index *k* denotes the iteration (or generation) number of the algorithm while the lower and upper parameter limits are represented by *l*_*i*_ and *u*_*i*_, respectively. The initial population, *S*_*k*_ = {*x*_1,*k*_, *x*_2,*k*_,…, *x*_*N*,*k*_} (being *k* = 1), is taken from uniformly distributed samples of the search region *X*. We also denote the population set at the *k*th iteration by *S*_*k*_. It does contain the members of the set *S*_*k*_ that have changed with *k*. After the initialization of *S*_*k*_, EMO continues its iterative process until a stopping condition (e.g., the maximum number of iterations) is met. An iteration of EMO consists of two steps. In the first step, each point in *S*_*k*_ moves to a different location by using the attraction-repulsion mechanism of the electromagnetism theory [21]. In the second step, points that have been moved by the electromagnetism theory are further moved locally by a local search and then become the members of *S*_*k*+1_ in the (*k* + 1)th iteration. Both the attraction-repulsion mechanism and the local search in EMO are responsible for driving the members, *x*_*i*,*k*_, of *S*_*k*_ to the close proximity of the global minimizer.

Similar to the electromagnetism theory for charged particles, each point *x*_*i*,*k*_ ∈ *S*_*k*_ in the search space *X* is assumed to be a charged particle where the charge of a point relates to its objective function value. Points holding better objective function values possess higher EMO charges than other points.

The attraction-repulsion mechanism in EMO states that points holding more charge attract other points in *S*_*k*_, while points showing less charge repel other points. Finally, a total force vector, *F*_*i*_^*k*^, exerted over a point, for example, the *i*th point *x*_*i*,*k*_, is calculated by adding the resultant attraction-repulsion forces, and each *x*_*i*,*k*_ ∈ *S*_*k*_ is moved in the direction of its total force, denoting its location by *y*_*i*,*k*_.

A local search is used to explore the neighborhood of each *y*_*i*,*k*_. Considering a determined number of steps, known as *I*_*l*_, and a fixed neighbourhood search *δ*, the procedure iterates as follows. Point *y*_*i*,*k*_ is assigned to a temporary point *z*_*i*,*k*_ to store the initial information. Next, for a given coordinate *i*  (∈1,…, *n*), a random number is selected and combined with *δ* as a step length, which in turns moves the point *z*_*i*,*k*_ along the direction *d*. If point *z*_*i*,*k*_ observes a better performance within a set of *I*_*l*_ repetitions, point *y*_*i*,*k*_ is replaced by *z*_*i*,*k*_; otherwise *y*_*i*,*k*_ is held. Therefore, the members, *x*_*i*,*k*+1_ ∈ *S*_*k*+1_, of the (*k* + 1)th iteration are defined through the following equation:

(1) $$\begin{array}{c} x_{i,k+1}=\{\begin{array}{cc} z_{i,k}, & \text{if}\;f\left(z_{i,k}\right)<f\left(y_{i,k}\right),\\ y_{i,k}, & \text{otherwise}. \end{array} \end{array}$$

Algorithm 1 shows the general scheme of EMO. We also provide the description of each step as follows. 

*Input Parameter Values (Line 1)*. The EMO algorithm is run for *MAXITER* iterations. In the local search phase, *n* × *I*_*l*_ is the maximum number of locations *z*_*i*,*k*_, within a *δ* distance of *y*_*i*,*k*_, for each *i*.

*Initialize (Line 2)*. The points *x*_*i*,*k*_, *k* = 1, are selected uniformly distributed in *X*, considering lower *l*_*i*_ and upper *u*_*i*_ parameter limits, where *i* = 1, 2,…, *n*. The objective function values *f*(*x*_*i*,*k*_) are computed, and the best point

(2) $$\begin{array}{c} x_{k}^{B}=\arg\underset{x_{i,k\in S_{k}}}{\min}\left\{f\left(x_{i,k}\right)\right\} \end{array}$$

is also identified.

*Calculate Force (Line 4)*. In this step, a charged-like value (*q*_*i*,*k*_) is assigned to each point (*x*_*i*,*k*_). The charge *q*_*i*,*k*_ of *x*_*i*,*k*_ is dependent on *f*(*x*_*i*,*k*_), and points holding better objective function have more charge than others. The charges are computed as follows:

(3) $$\begin{array}{c} q_{i,k}=\exp\left(-n\frac{f\left(x_{i,k}\right)-f\left(x_{k}^{B}\right)}{\sum_{j=1}^{N}f\left(x_{j,k}\right)-f\left(x_{k}^{B}\right)}\right), \end{array}$$

where *x*_*k*_^*B*^ represents the best particle in the population (see (2)). Then, the force, *F*_*i*,*j*_^*k*^, between two points, *x*_*i*,*k*_ and *x*_*j*,*k*_, is calculated by using

(4) $$\begin{array}{c} F_{i,j}^{k}=\{\begin{array}{cc} \left(x_{j,k}-x_{i,k}\right)\frac{q_{i,k}\cdot q_{j,k}}{{||x_{j,k}-x_{i,k}||}^{2}} & \text{if}\;\;f\left(x_{i,k}\right)>f\left(x_{j,k}\right),\\ \left(x_{i,k}-x_{j,k}\right)\frac{q_{i,k}\cdot q_{j,k}}{{||x_{j,k}-x_{i,k}||}^{2}} & \text{if}\;\;f\left(x_{i,k}\right)\leq f\left(x_{j,k}\right). \end{array} \end{array}$$

The total force, *F*_*i*_^*k*^, corresponding to *x*_*i*,*k*_ is now calculated as

(5) $$\begin{array}{c} F_{i}^{k}=\sum_{j=1,j\neq i}^{N}F_{i,j}^{k}. \end{array}$$

*Moving Point x*_*i*,*k*_ *along F*_*i*_^*k*^ *(Line 5)*. In this step, each point *x*_*i*,*k*_, except for *x*_*k*_^*B*^, is moved along the total force vector *F*_*i*_^*k*^ by considering

(6) $$\begin{array}{c} x_{i,k}=x_{i,k}+\lambda \frac{F_{i}^{k}}{||F_{i}^{k}||}\left(\text{RNG}\right),\;i=1,2,\ldots ,N;\;i\neq B, \end{array}$$

where *λ* is a random number between 0 and 1, and RNG denotes the allowed range of movement towards the lower *l*_*i*_ or upper *u*_*i*_ bound for the corresponding dimension.

*Local Search (Line 6)*. For each *y*_*i*,*k*_, a maximum of *I*_*l*_ points are generated at each coordinate direction in *δ*, the neighborhood of *y*_*i*,*k*_. The process of generating local points is continued for each *y*_*i*,*k*_ until either a better *z*_*i*,*k*_ is found or the *I*_*l*_ trial is reached.

*Selection for the Next Iteration (Line 7)*. In this step, members *x*_*i*,*k*+1_ ∈ *S*_*k*+1_ are selected from *y*_*i*,*k*_ and *z*_*i*,*k*_ using (1) and the best point is identified using (2).

## 3. Circle Detection Using EMO

### 3.1. Data Preprocessing

In order to detect circle shapes, candidate images must be preprocessed first by the well-known Canny algorithm which yields a single-pixel edge-only image. Then, the (*x*_*i*_, *y*_*i*_) coordinates for each edge pixel *p*_*i*_ are stored inside the edge vector *P* = {*p*_1_, *p*_2_,…, *p*_*N*_*p*__}, with *N*_*p*_ being the total number of edge pixels.

### 3.2. Particle Representation

**Figure 1:**
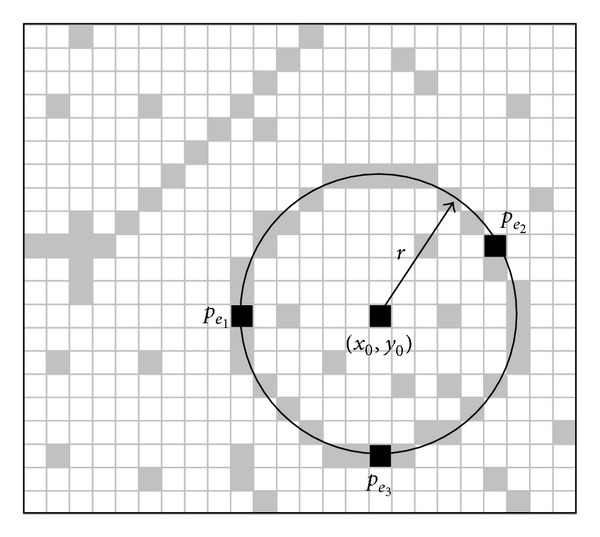
Circle candidate (charged particle) built from the combination of points *p*_*e*_1__,*p*_*e*_2__, and *p*_*e*_3__.

In order to construct each particle *C* (circle candidate), the indexes *e*_1_, *e*_2_, and *e*_3_, which represent three edge points previously stored inside the vector *P*, must be grouped assuming that the circle's contour connects them. Therefore, the circle *C* = {*p*_*e*_1__, *p*_*e*_2__, *p*_*e*_3__} passing over such points may be considered as a potential solution for the detection problem. Considering the configuration of the edge points shown in Figure 1, the circle center (*x*_0_, *y*_0_) and the radius *r* of *C* can be characterized as follows:

(7) $$\begin{array}{c} {\left(x-x_{0}\right)}^{2}+{\left(y-y_{0}\right)}^{2}=r^{2}, \end{array}$$

where *x*_0_ and *y*_0_ are computed through the following equations:

(8) x0=det⁡(A)4((xe2−xe1)(ye3−ye1)−(xe3−xe1)(ye2−ye1)),y0=det⁡⁡(B)4((xe2−xe1)(ye3−ye1)−(xe3−xe1)(ye2−ye1)),

with det⁡(**A**) and det⁡(**B**) representing determinants of matrices **A** and **B**; respectively; considering:

(9) $$\begin{array}{c} A=\left[\begin{array}{cc} x_{e_{2}}^{2}+y_{e_{2}}^{2}-\left(x_{e_{1}}^{2}+y_{e_{1}}^{2}\right) & 2\cdot \left(y_{e_{1}}-y_{e_{1}}\right)\\ x_{e_{3}}^{2}+y_{e_{3}}^{2}-\left(x_{e_{1}}^{2}+y_{e_{1}}^{2}\right) & 2\cdot \left(y_{e_{3}}-y_{e_{1}}\right) \end{array}\right],\\ B=\left[\begin{array}{cc} 2\cdot \left(x_{e_{2}}-x_{e_{1}}\right) & x_{e_{2}}^{2}+y_{e_{2}}^{2}-\left(x_{e_{1}}^{2}+y_{e_{1}}^{2}\right)\\ 2\cdot \left(x_{e_{3}}-x_{e_{1}}\right) & x_{e_{3}}^{2}+y_{e_{3}}^{2}-\left(x_{e_{1}}^{2}+y_{e_{1}}^{2}\right) \end{array}\right], \end{array}$$

the radius *r* can therefore be calculated using

(10) $$\begin{array}{c} r=\sqrt{{\left(x_{0}-x_{e_{d}}\right)}^{2}+{\left(y_{0}-y_{e_{d}}\right)}^{2}}, \end{array}$$

where *d* ∈ {1,2, 3}, and (*x*_*e*_*d*__, *y*_*e*_*d*__) are the coordinates of any of the three selected points which define the particle *C*. Figure 1 illustrates main parameters defined by (7)–(10). Therefore, the shaping parameters for the circle [*x*_0_,*y*_0_, *r*] can be represented as a transformation *T* of the edge vector indexes *e*_1_, *e*_2_, and *e*_3_:

(11) $$\begin{array}{c} \left[x_{0},y_{0},r\right]=T\left(e_{1},e_{2},e_{3}\right). \end{array}$$

By exploring each index as a particle, it is possible to sweep the continuous space while looking for shape parameters by means of the EMO algorithm. This approach reduces the search space by eliminating unfeasible solutions.

### 3.3. Objective Function

In order to model the fitness function, the circumference coordinates of the circle candidate *C* are calculated as a virtual shape which, in turn, must be validated, that is, if it really exists in the edge image. The circumference coordinates are grouped within the test set *H* = {*h*_1_, *h*_2_,…, *h*_*N*_*s*__}, with *N*_*s*_ representing the number of points over which the existence of an edge point, which corresponds to *C*, must be verified.

The test *H* is generated by the midpoint circle algorithm (MCA) [22] which is a well-known algorithm to determine the required points for drawing a circle. MCA requires as inputs only the radius *r* and the center point (*x*_0_, *y*_0_) considering only the first octant over the circle equation *x* ^2^ + *y* ^2^ = *r* ^2^. It draws a curve starting at point (*r*, 0) and proceeds upwards left by using integer additions and subtractions. The MCA aims to calculate the required points *H* in order to represent a circle candidate. Although the algorithm is considered as the quickest providing a subpixel precision, it is important to assure that points lying outside the image plane must not be considered in *H*.

The objective function *J*(*C*) represents the matching error produced between the pixels *H* of the circle candidate *C* (particle) and the pixels that actually exist in the edge-only image, yielding

(12) $$\begin{array}{c} J\left(C\right)=1-\frac{\sum_{v=1}^{N_{s}}E\left(h_{v}\right)}{N_{s}}, \end{array}$$

where *E*(*h*_*v*_) is a function that verifies the pixel existence in *h*_*v*_, where *h*_*v*_ ∈ *H* and *N*_*s*_ is the number of elements of *H*. Hence the function *E*(*h*_*v*_) is defined as

(13) $$\begin{array}{c} E\left(h_{v}\right)=\{\begin{array}{cc} 1, & \text{if}\;\text{the}\;\text{test}\;\text{pixel}\;h_{v}\;\;\text{is}\;\text{an}\;\text{edge}\;\text{point},\\ 0, & \text{otherwise}. \end{array} \end{array}$$

**Figure 2:**
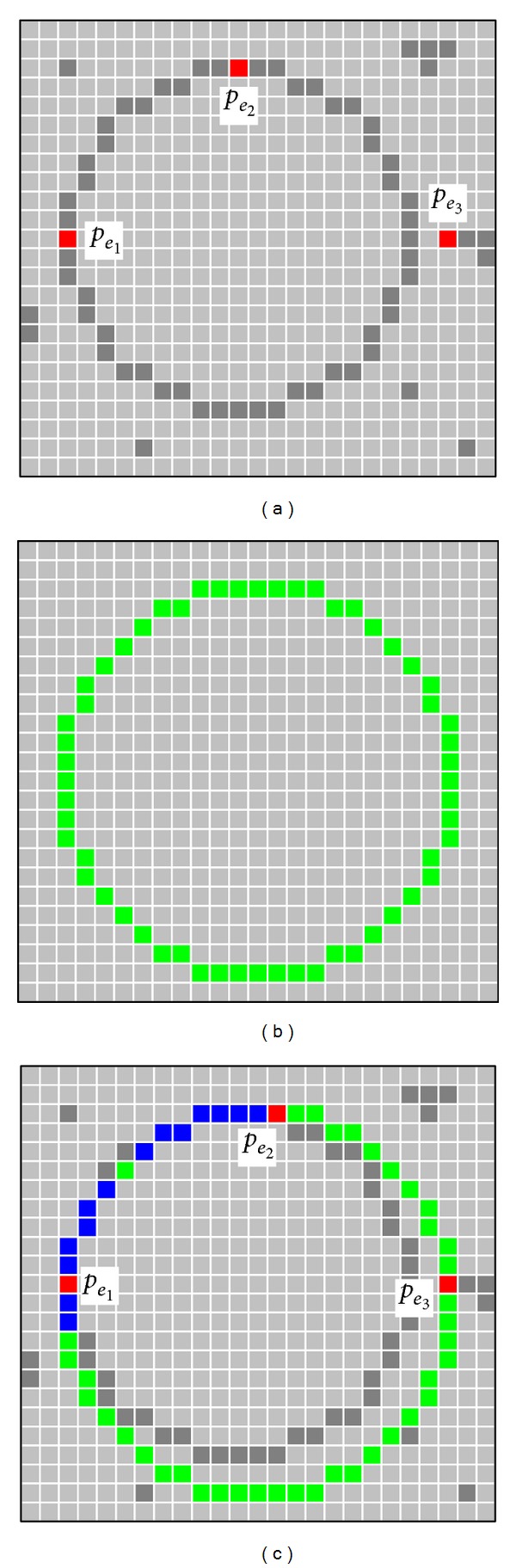
Procedure to evaluate the objective function *J*(*C*). The image shown by (a) presents the original edge image while (b) portraits the virtual shape *H* corresponding to *C*. The image in (c) shows coincidences between both images through blue or red pixels while the virtual shape is also depicted in green.

A value of *J*(*C*) near to zero implies a better response from the “circularity” operator.  Figure 2 shows the procedure to evaluate a candidate solution *C* with its representation as a virtual shape *H*.  Figure 2(a) shows the original edge map, while Figure 2(b) presents the virtual shape *H* representing the particle *C* = {*p*_*e*_1__, *p*_*e*_2__, *p*_*e*_3__}. In Figure 2(c), the virtual shape *H* is compared to the original image, point by point, in order to find coincidences between virtual and edge points. The *p* individual has been built from points *p*_*e*_1__, *p*_*e*_2__, and *p*_*e*_3__ which are shown in Figure 2(a). The virtual shape *H*, obtained by MCA, gathers 56 points (*N*_*s*_ = 56) with only 18 of them existing in both images (shown as blue points plus red points in Figure 2(c)) and yielding; ∑_*h*=1_^*N*_*s*_^ *E*(*h*_*v*_) = 18; therefore *J*(*C*) ≈ 0.67.

### 3.4. EMO Implementation

The implementation of the proposed algorithm can be summarized into the following steps.

**Step 1**

The Canny filter is applied to find the edges and store them in the *P* = {*p*_1_, *p*_2_,…, *p*_*N*_*p*__} vector. The index *k* is set to 1.

**Step 2**

*m* initial particles are generated (*C*_*a*,1_, *a* ∈ [1, *m*]). Particles belonging to a seriously small or to a quite big radius are eliminated (collinear points are discarded).

**Step 3**

The objective function *J*(*C*_*a*,*k*_) is evaluated to determine the best particle *C* ^*B*^ (where *C* ^*B*^ ← arg min⁡{*J*(*C*_*a*,*k*_)}).

**Step 4**

The charge between particles is calculated using expression (3), and its vector force is calculated through (4) and (5). The particle with a better objective function holds a bigger charge and therefore a bigger attraction force.

**Step 5**

The particles are moved according to their force magnitude. The new particle's position *C*_*a*_^*y*^ is calculated by expression (6). *C* ^*B*^ is not moved because it has the biggest force and it attracts other particles to itself.

**Step 6**

For each *C*_*a*_^*y*^, a maximum of *I*_*l*_ points are generated at each coordinate direction in the *δ* neighborhood of *C*_*a*_^*y*^. The process of generating local points is continued for each *C*_*a*_^*y*^ until either a better *C*_*a*_^*z*^ is found or the *n* × *I*_*l*_ trial is reached.

**Step 7**

The new particles *C*_*a*,*k*+1_ are selected from *C*_*a*_^*y*^ and *C*_*a*_^*z*^ using (1).

**Step 8**

The *k* index is increased. If *k* = *MAXITER* or if *J*(*C*_*a*,*k*_) value is as smaller as the predefined threshold value, then the algorithm is stopped and the flow jumps to Step 9. Otherwise, it jumps to Step 3.

**Step 9**

The best *C* ^*B*^ particle is selected from the last iteration.

**Step 10**

From the original edge map, the algorithm marks points corresponding to *C* ^*B*^. In case of multicircle detection, it jumps to Step 2.

**Step 11**

Finally, the best particle *C*_*Nc*_^*B*^ from each circle is used to draw (over the original image) the detected circles, considering *Nc* as the number of detected circles.

**Figure 3:**
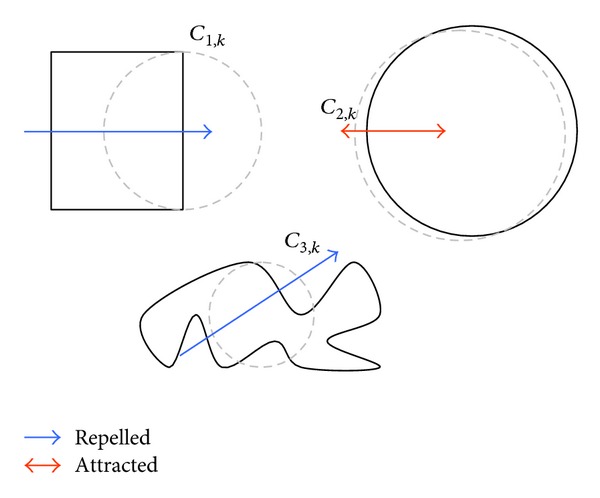
An analogy to the Coulomb's law.

Figure 3 shows an analogy to Coulomb's law. The original figures to be detected are represented by a solid black line while the shapes with discontinuous gray lines represent the candidate circles. Since the candidate circles *C*_1,*k*_ and *C*_3,*k*_ present a high value in the fitness function *J*(*C*_*a*,*k*_), they are repelled (blue lines), moving away the shapes. In contrast, the circle candidate *C*_2,*k*_ that holds a small value of *J*(*C*_*a*,*k*_) is attracted (red line) to the circular shape contained in the image. 

## 4. The White Blood Cell Detector

In order to detect WBC, the proposed detector combines the EMO-based circle detector presented in Section 3 with a new objective function.

### 4.1. Image Preprocessing

To employ the proposed detector, smear images must be preprocessed to obtain two new images: the segmented image and its corresponding edge map. The segmented image is produced by using a segmentation strategy whereas the edge map is generated by a border extractor algorithm. Both images are considered by the new objective function to measure the resemblance of a candidate circle with an actual WBC.

The goal of the segmentation strategy is to isolate the white blood cells (WBCs) from other structures such as red blood cells and background pixels. Information of color, brightness, and gradients is commonly used within a thresholding scheme to generate the labels to classify each pixel. Although a simple histogram thresholding can be used to segment the WBCs, in this work, the diffused expectation-maximization (DEM) has been used to assure better results [23].

DEM is an expectation-maximization- (EM-) based algorithm which has been used to segment complex medical images [24]. In contrast to classical EM algorithms, DEM considers the spatial correlations among pixels as a part of the minimization criteria. Such adaptation allows to segment objects in spite of noisy and complex conditions.

**Figure 4:**
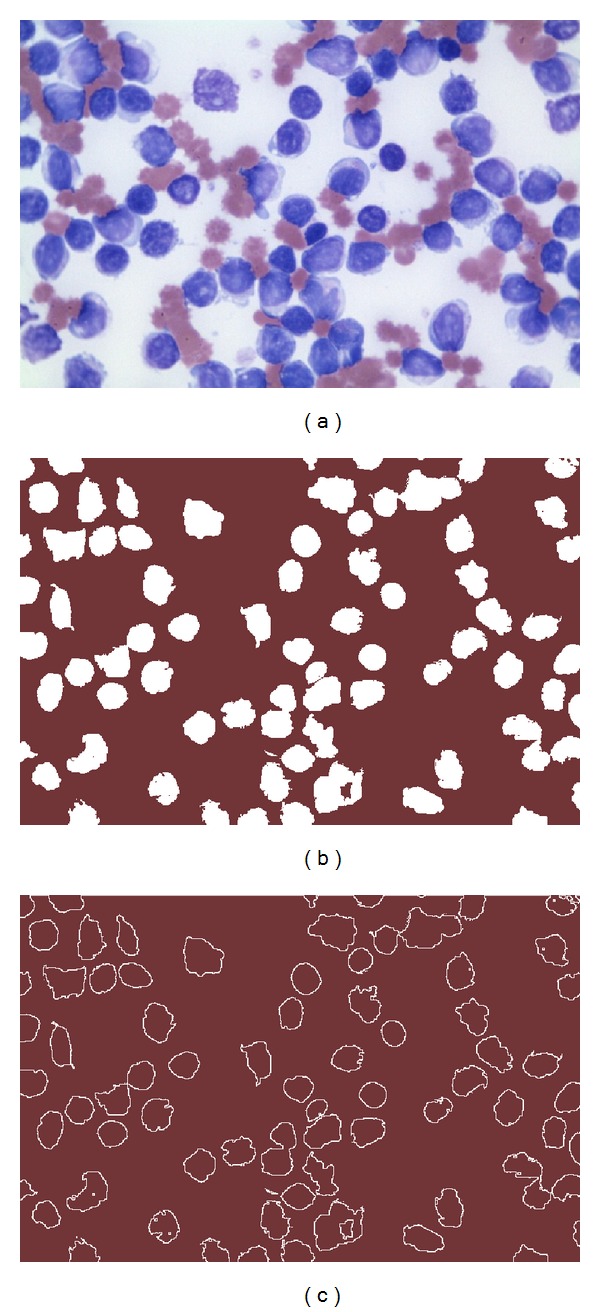
Preprocessing process: (a) original smear image, (b) segmented image obtained by DEM, and (c) the edge map obtained by using the morphological edge detection procedure.

For the WBCs segmentation, the DEM has been configured considering three different classes (*K* = 3), *g*(∇*h*_*ik*_) = |∇*h*_*ik*_|^−9/5^, *λ* = 0.1, and *m* = 10 iterations. These values have been found as the best configuration set according to [23]. As a final result of the DEM operation, three different thresholding points are obtained: the first corresponds to the WBCs, the second to the red blood cells, whereas the third represents the pixels classified as background.  Figure 4(b) presents the segmentation results obtained by the DEM approach employed at this work considering Figure 4(a) as the original image.

Once the segmented image has been produced, the edge map is computed. The purpose of the edge map is to obtain a simple image representation that preserves object structures. Optimization-based circle detectors [17–20] operate directly over the edge map in order to recognize circular shapes. Several algorithms can be used to extract the edge map; however, in this work, the morphological edge detection procedure [25] has been used to accomplish such a task. Morphological edge detection is a traditional method to extract borders from binary images in which original images (*I*_*B*_) are eroded by a simple structure element (*I*_*E*_). Then, the eroded image is inverted (${\overset{-}{I}}_{E}$) and compared with the original image (${\overset{-}{I}}_{E}\wedge I_{B}$) in order to detect pixels which are present in both images. Such pixels compose the computed edge map from *I*_*B*_.  Figure 4(c) shows the edge map obtained by using the morphological edge detection procedure.

Other example is presented in Figure 8. It represents a complex example with an image showing seriously deformed cells. Despite such imperfections, the proposed approach can effectively detect the cells as it is shown in Figure 8(d). 

### 4.2. The Modified EMO-Based Circle Detector

The circle detection approach uses the encoding of three edge points that represent candidate circles in the image. In the original EMO-based circle detector, the quality of each individual is calculated by using an objective function which evaluates the existence of a candidate circle considering only information from the edge map (shape structures). The better a candidate circle approximates the actual edge-circle, the more the objective function value decreases. Therefore, the detection performance depends on the quality of the edge map that is obtained from the original images. However, since smear images present different imaging conditions and staining intensities, they produce edge maps partially damaged by noisy pixels. Under such conditions, the use of the EMO-based circle detector cannot be directly applied to WBC detection.

In order to use the EMO-based circle detector within the context of WBC detection, it is necessary to change the fitness function presented in (11). In this work, a new objective function has been derived to measure the resemblance of a candidate circle to an actual WBC based on the information from the edge map and the segmented image. Such new objective function takes into consideration not only the information provided by the edge map but also the relationship among the pixels falling inside the candidate circle which is contained in the segmented image, validating the existence of the WBC. This new function *J*(*C*) is thus calculated as follows:

(14) $$\begin{array}{c} J_{\text{New}}\left(C\right)=2-\frac{\sum_{v=1}^{N_{s}}E\left(h_{v}\right)}{N_{s}}-\frac{Wp}{Bp}, \end{array}$$

where *h*_*v*_ and *N*_*s*_ keep the same meaning than (11) and *Wp* is the amount of white pixel falling inside the candidate circle represented by *C*. Likewise, *Bp* corresponds to the total number of black pixels falling inside *C*.

**Figure 5:**
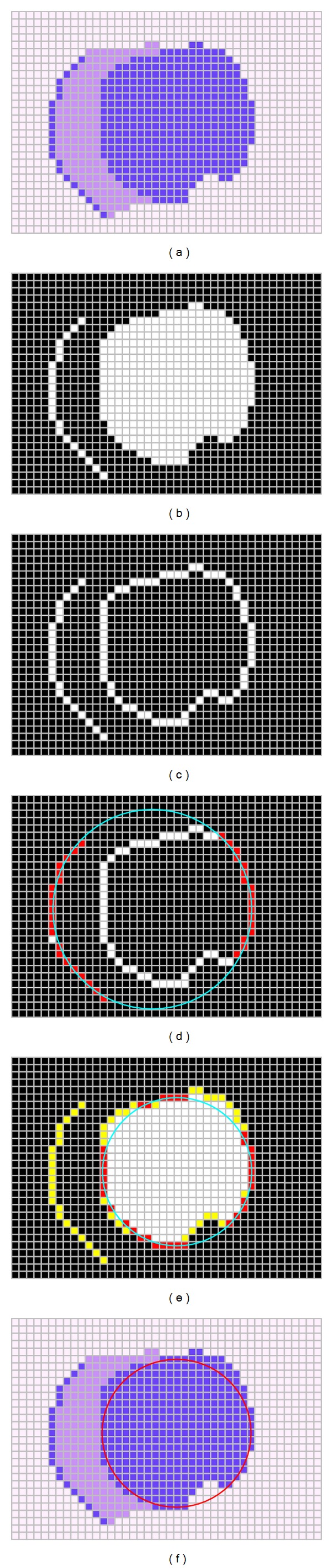
WBC detection procedure. (a) Smear image. (b) Segmented image. (c) Edge map. (d) Detected circle by using the original objective function. Red points show the coincidences between the candidate circle and the edge map. (e) Detected circle by using the new objective function. Yellow points represent the edge pixels without coincidence. (f) Final result.

To illustrate the functionality of the new objective function, Figure 5 presents a detection procedure which considers a complex image.  Figure 5(a) shows the original smear image containing a WBC and a stain produced by the coloring process. Figures 5(b) and 5(c) represent the segmented image and the edge map, respectively. Since the stain contained in the smear image (Figure 5(a)) possesses similar properties than a WBC, it remains as a part of the segmented image (Figure 5(b)) and the edge map (Figure 5(c)). Such an inconsistency produces big detection errors in case the EMO-based circle detector is used without modification.  Figure 5(d) presents detection results obtained by the original EMO-based circle detector. As the original objective function considers only the number of coincidences between the candidate circle and the edge map, circle candidates that match with a higher number of edge pixels are chosen as the best circle instances. In Figure 5(d), the detected circle presents a coincidence of 37 different pixels in the edge map. Such coincidence is considered as the best possible under the restrictions of the original objective function. On the other hand, when the modified objective function is used in the recognition procedure, the accuracy and the robustness of the detection are both significantly improved. By using the new objective function, information from the segmented image is employed to refine the solution that is provided by coincidences with the edge map.  Figure 5(e) presents the detection result that has been produced by the modified EMO-based circle detector. In the figure, the detected circle matches with only 32 pixels of the edge map. However, it is considered as the best instance due to the relationship of its internal pixels (the white pixels are much more than the black pixels). Finally, Figure 5(f) shows final detection results over the original smear image.

Table 1 presents the parameters for the EMO algorithm used in this work. They have been kept for all test images after being experimentally defined.

Under such assumptions, the complete process to detect WBCs is implemented as follows.

**Step 1**

Segment the WBCs using the DEM algorithm.

**Step 2**

Get the edge map from the segmented image by using the morphological edge detection method.

**Step 3**

Start the circle detector based on EMO over the edge map while saving best circles (Section 3.3).

**Step 4**

Define parameter values for each circle that identify the WBCs.

### 4.3. Numerical Example

**Figure 6:**
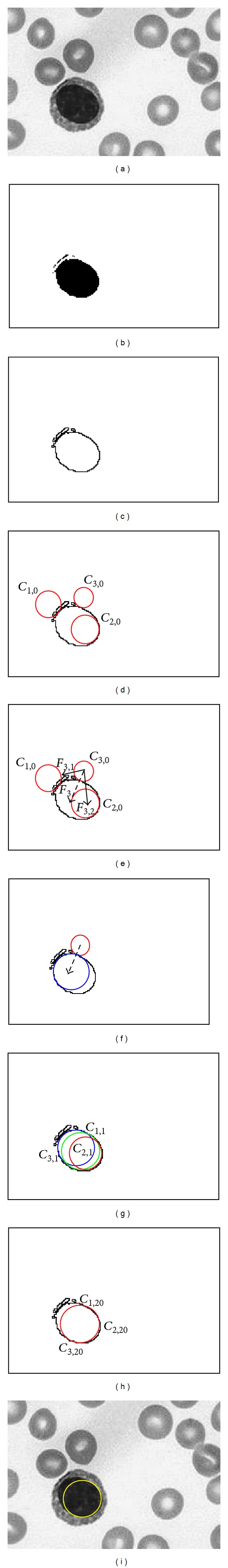
Detection numerical example: (a) The image used as example. (b) Segmented image. (c) Edge map. (d) Initial particles. (e) Forces exerted over *C*_3,0_. (f) New position of *C*_3,0_. (g) Positions of all particles after the first generation. (h) Final particle configuration after 20 generations. (i) Final result overlapped the original image.

In order to present the algorithm's step-by-step operation, a numerical example has been set by applying the proposed method to detect a single leukocyte lying inside of a simple image.  Figure 6(a) shows the image used in the example. After applying the threshold operation, the WBC is located besides few other pixels which are merely noise (see Figure 6(b)). Then, the edge map is subsequently computed and stored pixel by pixel inside the vector *P*.  Figure 6(c) shows the resulting image after such procedure.

The EMO-based circle detector is executed using information of the edge map and the segmented image (for the sake of easiness, it only considers a population of three particles). Like all evolutionary approaches, EMO is a population-based optimizer that attacks the starting point problem by sampling the search space at multiple, randomly chosen, initial particles. By taking three random pixels from vector *P*, three different particles are constructed.  Figure 6(d) depicts the initial particle distribution. Since the particle *C*_2,0_ holds the best fitness value *J*_New_(*C*_2,0_) (it does possess a better coincidence with the edge map and a god pixel relationship), it is considered as the best particle *C* ^*B*^. Then, the charge of each particle is calculated through (3), and the forces exerted over each particle are computed.  Figure 6(e) shows the forces exerted over the *C*_3,0_ particle. Since the *C*_3,0_ particle is the worst particle in terms of fitness value, it is attracted by particles *C*_1,0_ and *C*_2,0_. *F*_3,1_ and *F*_3,2_ represent the existent attracting forces of *C*_3,0_ with respect to *C*_1,0_ and *C*_2,0_ whereas *F*_3_ corresponds to the resultant force. Considering *F*_3_ as the final force exerted over *C*_3,0_, the position of *C*_3,0_ is modified using (6).  Figure 6(f) depicts the new position *C*_3,1_ of particle *C*_3,0_ (the second subindex means the iteration number). If the same procedure is applied over all the particles (except for *C*_2,0_ which is the best particle), it yields positions shown in Figure 6(g). Therefore, after 20 iterations, all particles converge to the same position presented in Figure 6(h) whereas Figure 6(i) depicts the final result.

## 5. Experimental Results

Experimental tests have been developed in order to evaluate the performance of the WBC detector. It was tested over microscope images from blood smears holding a 600 × 500 pixel resolution. They correspond to supporting images on the leukemia diagnosis. The images show several complex conditions such as deformed cells and overlapping with partial occlusions. The robustness of the algorithm has been tested under such demanding conditions. 

**Figure 7:**
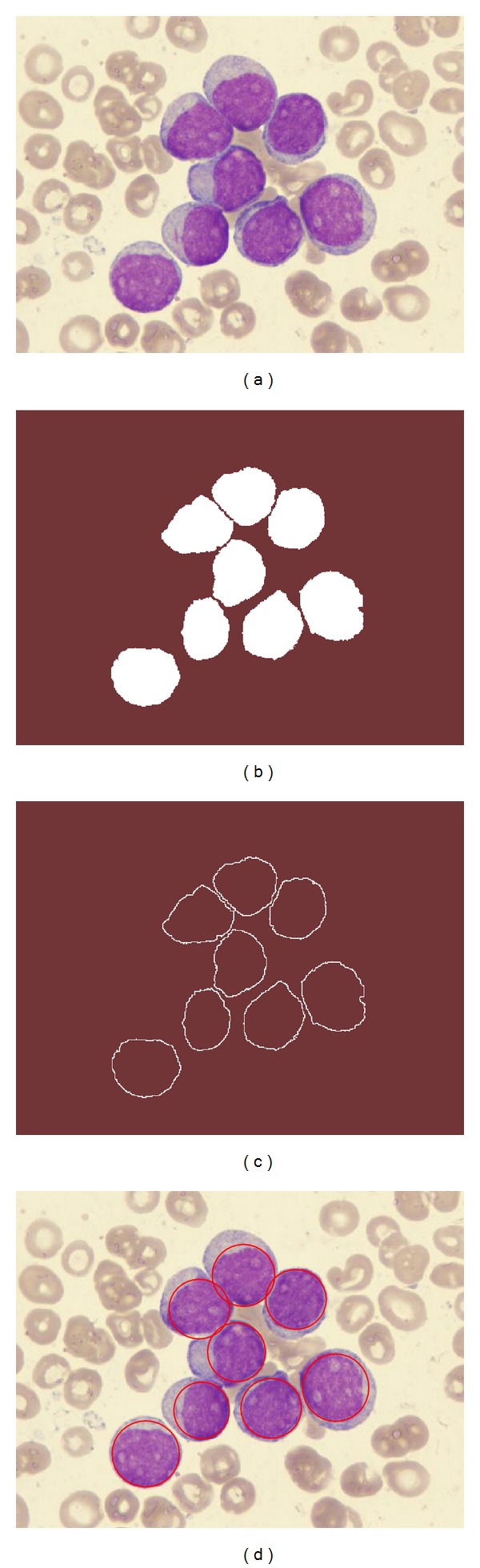
Resulting images of the first test after applying the WBC detector: (a) original image, (b) image segmented by the DEM algorithm, (c) edge map, and (d) the white detected blood cells.

**Figure 8:**
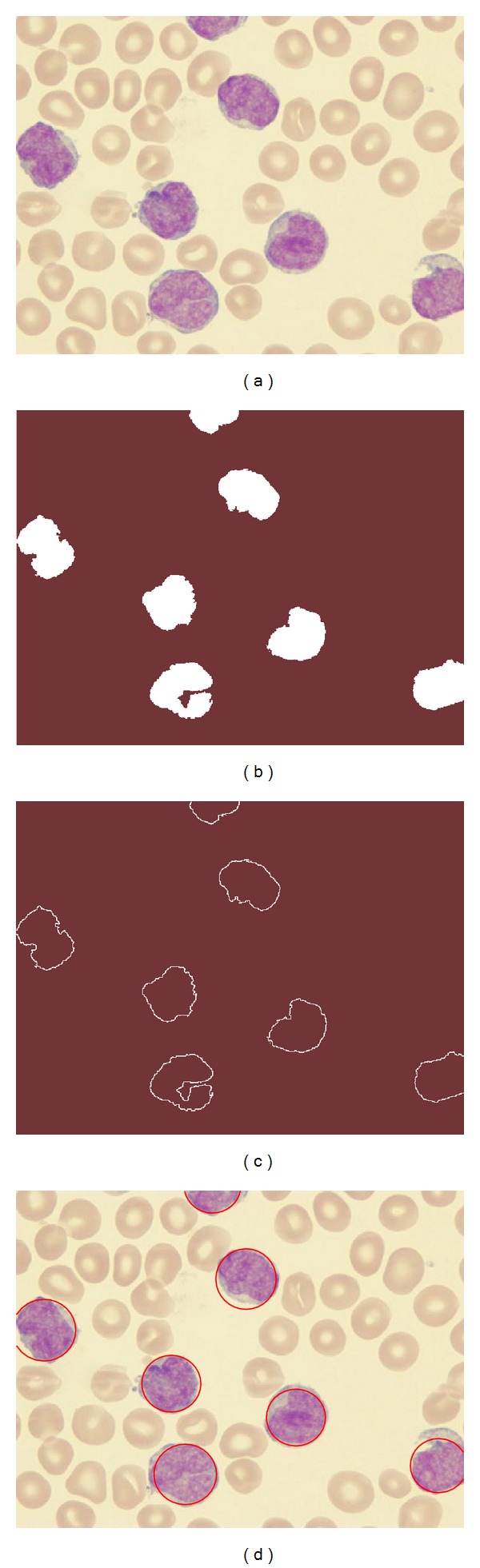
Resulting images of the second test after applying the WBC detector: (a) original image, (b) image segmented by the DEM algorithm, (c) edge map, and (d) the white detected blood cells.

Figure 7(a) shows an example image employed in the test. It was used as input image for the WBC detector.  Figure 7(b) presents the segmented WBCs obtained by the DEM algorithm. Figures 7(c) and 7(d) present the edge map and the white blood cells after detection, respectively. The results show that the proposed algorithm can effectively detect and mark blood cells despite cell occlusion, deformation or overlapping. Other parameters may also be calculated through the algorithm: the total area covered by white blood cells and relationships between several cell sizes.

## 6. Comparisons to Other Methods

A comprehensive set of smear-blood test images is used to test the performance of the proposed approach. We have applied the proposed EMO-based detector to test images in order to compare its performance to other WBC detection algorithms such as the boundary support vectors (BSVs) approach [2], the iterative Otsu (IO) method [3], the Wang algorithm [4], and the Genetic algorithm-based (GAB) detector [17]. In all cases, the algorithms are tuned according to the value set which is originally proposed by their own references.

### 6.1. Detection Comparison

To evaluate the detection performance of the proposed detection method, Table 2 tabulates the comparative leukocyte detection performance of the BSV approach, the IO method, the Wang algorithm, the BGA detector and the proposed method, in terms of detection rates and false alarms. The experimental data set includes 30 images which are collected from the Cellavision reference library (http://www.cellavision.com). Such images contain 426 leukocytes (222 bright leukocytes and 204 dark leukocytes according to smear conditions) which have been detected and counted by a human expert. Such values act as ground truth for all the experiments. For the comparison, the detection rate (DR) is defined as the ratio between the number of leukocytes correctly detected and the number of leukocytes determined by the expert. The false alarm rate (FAR) is defined as the ratio between the number of nonleukocyte objects that have been wrongly identified as leukocytes and the number leukocytes which have been actually determined by the expert.

Experimental results show that the proposed EMO method, which achieves 96.48% leukocyte detection accuracy with 3.75% false alarm rate, is compared favorably against other WBC detection algorithms, such as the BSV approach, the IO method, the Wang algorithm, and the BGA detector.

### 6.2. Robustness Comparison

**Figure 9:**
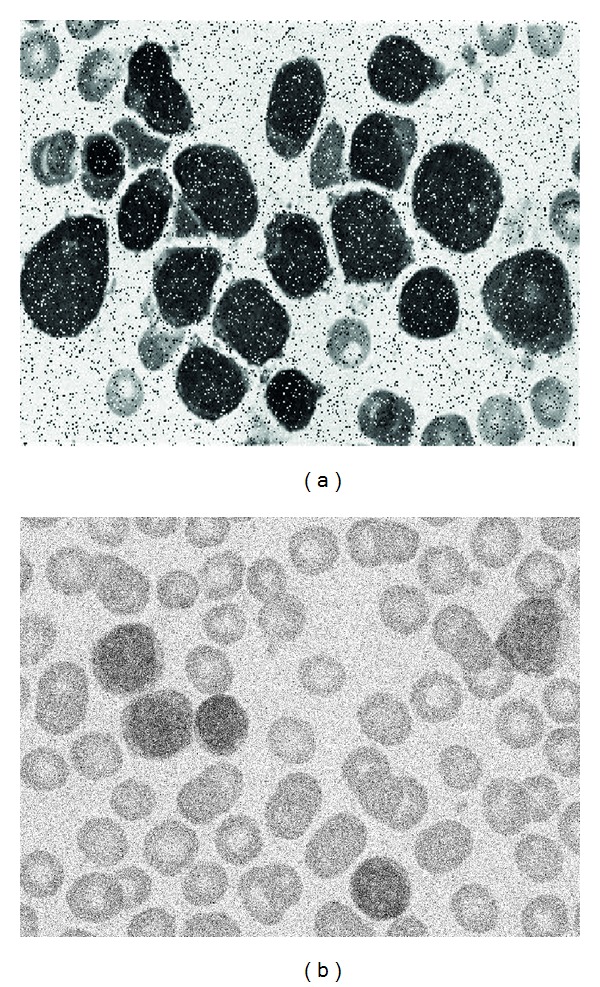
Examples of images included in the experimental set for robustness comparison. (a) Image contaminated with 10% of salt and pepper noise and (b) image polluted with *σ* = 10 of Gaussian noise.

Images of blood smear are often deteriorated by noise due to various sources of interference and other phenomena that affect the measurement processes in imaging and data acquisition systems. Therefore, the detection results depend on the algorithm's ability to cope with different kinds of noises. In order to demonstrate the robustness in the WBC detection, the proposed EMO approach is compared to the BSV approach, the IO method, the Wang algorithm and the BGA detector under noisy environments. In the test, two different experiments have been studied. The first inquest explores the performance of each algorithm when the detection task is accomplished over images corrupted by salt and pepper noise. The second experiment considers images polluted by Gaussian noise. Salt and Pepper and Gaussian noise are selected for the robustness analysis because they represent the most compatible noise types commonly found in images of blood smear [26]. The comparison considers the complete set of 30 images presented in Section 6.1 containing 426 leukocytes which have been detected and counted by a human expert. The added noise is produced by MatLab, considering two noise levels of 5% and 10% for salt and pepper noise whereas *σ* = 5 and *σ* = 10 are used for the case of Gaussian noise.  Figure 9 shows only two images with different noise types as example. The outcomes in terms of the detection rate (DR) and the false alarm rate (FAR) are reported for each noise type in Tables 3 and 4. The results show that the proposed EMO algorithm presents the best detection performance, achieving in the worst case a DR of 87.79% and 89.20%, under contaminated conditions of salt and pepper and Gaussian noise, respectively. On the other hand, the EMO detector possesses the least degradation performance presenting a FAR value of 8.21% and 7.51%.

### 6.3. Stability Comparison

In order to compare the stability performance of the proposed method, its results are compared to those reported by Wang et al. in [4] which is considered as an accurate technique for the detection of WBC.

The Wang algorithm is an energy-minimizing method which is guided by internal constraint elements and influenced by external image forces, producing the segmentation of WBCs at a closed contour. As external forces, the Wang approach uses edge information which is usually represented by the gradient magnitude of the image. Therefore, the contour is attracted to pixels with large image gradients, that is, strong edges. At each iteration, the Wang method finds a new contour configuration which minimizes the energy that corresponds to external forces and constraint elements.

In the comparison, the net structure and its operational parameters, corresponding to the Wang algorithm, follow the configuration suggested in [4] while the parameters for the EMO algorithm are taken from Table 1.

**Figure 10:**
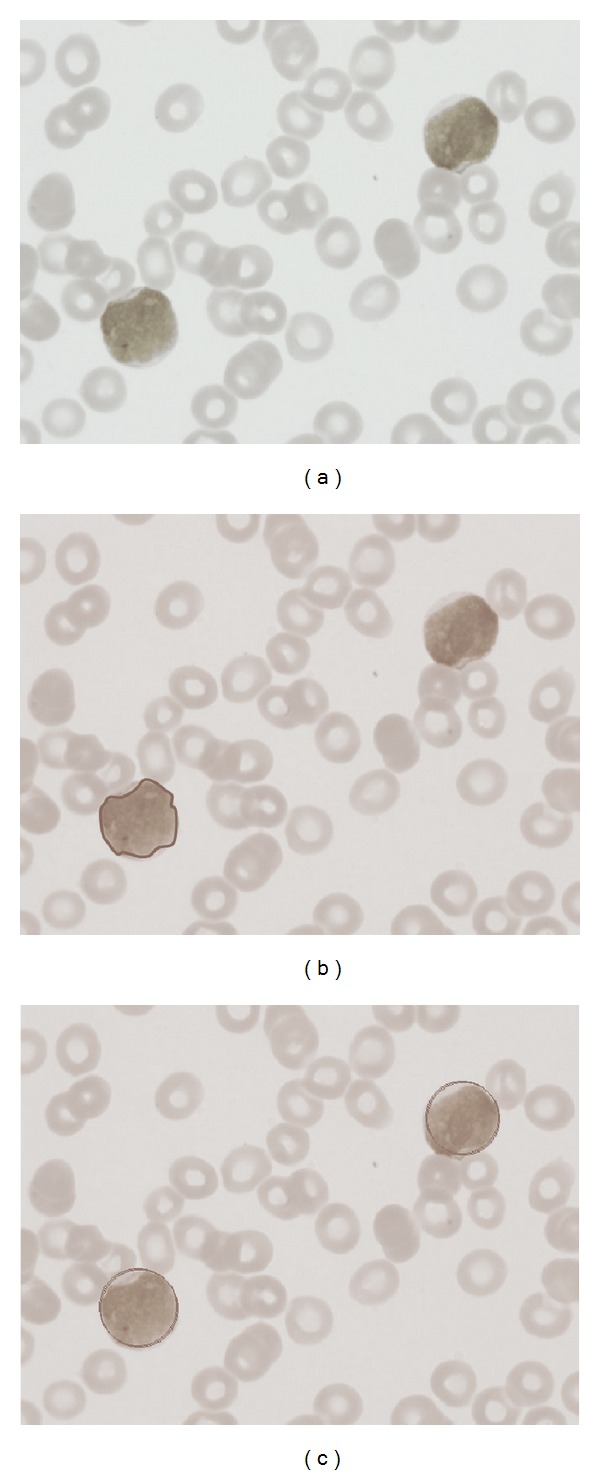
Comparison of the EMO and Wang's method for white blood cell detection in medical images. (a) Original image. (b) Detection using the Wang's method. (c) Detection after applying the EMO method.

Figure 10 shows the performance of both methods considering a test image with only two white blood cells. Since the Wang method uses gradient information in order to appropriately find a new contour configuration, it needs to be executed iteratively in order to detect each structure (WBC).  Figure 10(b) shows the results after the Wang approach has been applied considering only 200 iterations. Furthermore, Figure 10(c) shows results after applying the EMO method which has been proposed in this paper.

**Figure 11:**
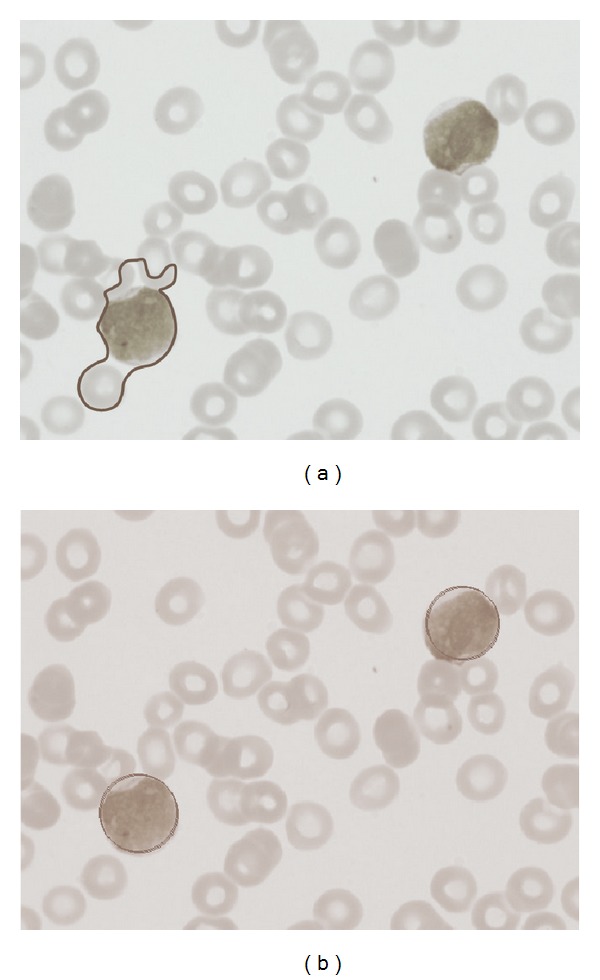
Result comparison for the white blood cells detection showing (a) Wang's algorithm after 400 cycles and (b) EMO detector method considering 1000 cycles.

The Wang algorithm uses the fuzzy cellular neural network (FCNN) as an optimization approach. It employs gradient information and internal states in order to find a better contour configuration. In each iteration, the FCNN tries, as contour points, different new pixel positions which must be located nearby the original contour position. Such fact might cause the contour solution to remain trapped into a local minimum. In order to avoid such a problem, the Wang method applies a considerable number of iterations so that a near optimal contour configuration can be found. However, when the number of iterations increases, the possibility to cover other structures increases too. Thus, if the image has a complex background (as smear images), the method gets confused so that finding the correct contour configuration from the gradient magnitude is not easy. Therefore, a drawback of Wang's method is related to its optimal iteration number (instability). Such number must be determined experimentally as it depends on the image context and its complexity.  Figure 11(a) shows the result of applying 400 cycles of Wang's algorithm while Figure 11(b) presents the detection of the same cell shapes after 1000 iterations using the proposed algorithm. From Figure 11(a), it can be seen that the contour produced by Wang's algorithm degenerates as the iteration process continues, wrongly covering other shapes lying nearby.

In order to compare the accuracy of both methods, the estimated WBC area, which has been approximated by both approaches, is compared to the actual WBC size considering different degrees of evolution, that is, the cycle number for each algorithm. The comparison acknowledges only one WBC because it is the only detected shape in Wang's method.  Table 5 shows the averaged results over twenty repetitions for each experiment.

## 7. Conclusions

This paper has presented an algorithm for the automatic detection of white blood cells that are embedded into complicated and cluttered smear images by considering the complete process as a circle detection problem. The approach is based on a nature-inspired technique called the electromagnetism-like optimization (EMO) which is a heuristic method that follows electromagnetism principles for solving complex optimization problems. The EMO algorithm is based on electromagnetic attraction and repulsion forces among charged particles whose charge represents the fitness solution for each particle (a given solution). The algorithm uses the encoding of three noncollinear edge points as candidate circles over an edge map. A new objective function has been derived to measure the resemblance of a candidate circle to an actual WBC based on the information from the edge map and segmentation results. Guided by the values of such objective function, the set of encoded candidate circles (charged particles) are evolved by using the EMO algorithm so that they can fit into the actual blood cells that are contained in the edge map.

The performance of the EMO-method has been compared to other existing WBC detectors (the boundary support vectors (BSV) approach [2], the iterative Otsu (IO) method [3], the Wang algorithm [4], and the Genetic algorithm-based (GAB) detector [19]) considering several images which exhibit different complexity levels. Experimental results demonstrate the high performance of the proposed method in terms of detection accuracy, robustness, and stability.

## Figures and Tables

**Algorithm 1:**
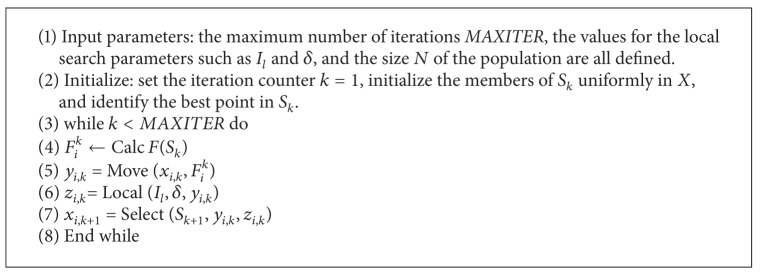
[EMO (*N*, *MAXITER*, *I*_*l*_, *δ*)].

**Table 1:** EMO parameters used for leukocytes detection in medical images.

*m*	*n*	*MA* *XI* *TE* *R*	*δ*	*LI* *S* *TE* *R*
50	3	5	4	4

**Table 2:** Comparative leukocyte detection performance of the BSV approach, the IO method, the Wang algorithm, the BGA detector, and the proposed EMO method over the data set which contains 30 images and 426 leukocytes.

Leukocyte type	Method	Leukocytes detected	Missing	False alarms	DR	FAR
Bright leukocytes (222)	BSV	104	118	67	46.85%	30.18%
	IO	175	47	55	78.83%	24.77%
	Wang	186	36	42	83.78%	18.92%
	BGA	177	45	22	79.73%	9.91%
	EMO	211	11	10	95.04%	4.50%

Dark leukocytes (204)	BSV	98	106	54	48.04%	26.47%
	IO	166	38	49	81.37%	24.02%
	Wang	181	23	38	88.72%	18.63%
	BGA	170	34	19	83.33%	9.31%
	EMO	200	4	6	98.04%	2.94%

Overall (426)	BSV	202	224	121	47.42%	28.40%
	IO	341	85	104	80.05%	24.41%
	Wang	367	59	80	86.15%	18.78%
	BGA	347	79	41	81.45%	9.62%
	EMO	411	15	16	96.48%	3.75%

**Table 3:** Comparative WBC detection among methods that considers the complete data set of 30 images corrupted by different levels of salt and pepper noise.

Noise level	Method	Leukocytes detected	Missing	False alarms	DR	FAR
5% salt and pepper noise 426 leukocytes	BSV	148	278	114	34.74%	26.76%
	IO	270	156	106	63.38%	24.88%
	Wang	250	176	118	58.68%	27.70%
	BGA	306	120	103	71.83%	24.18%
	EMO	390	36	30	91.55%	7.04%

10% salt and pepper noise 426 leukocytes	BSV	101	325	120	23.71%	28.17%
	IO	240	186	78	56.34%	18.31%
	Wang	184	242	123	43.19%	28.87%
	BGA	294	132	83	69.01%	19.48%
	EMO	374	52	35	87.79%	8.21%

**Table 4:** Comparative WBC detection among methods that considers the complete data set of 30 images corrupted by different levels of Gaussian noise.

Noise level	Method	Leukocytes detected	Missing	False alarms	DR	FAR
*σ* = 5 Gaussian noise 426 Leukocytes	BSV	172	254	77	40.37%	18.07%
	IO	309	117	71	72.53%	16.67%
	Wang	301	125	65	70.66%	15.26%
	BGA	345	81	61	80.98%	14.32%
	EMO	397	29	21	93.19%	4.93%

*σ* = 10 Gaussian noise 426 Leukocytes	BSV	143	283	106	33.57%	24.88%
	IO	281	145	89	65.96%	20.89%
	Wang	264	162	102	61.97%	23.94%
	BGA	308	118	85	72.30%	19.95%
	EMO	380	46	32	89.20%	7.51%

**Table 5:** Error in cell's size estimation after applying the EMO algorithm and the Wang's method to detect one leukocite embedded into a blood-smear image. The error is averaged over twenty experiments.

Algorithm	Iterations	Error %
Wang	60	70%
	200	1%
	400	121%

EMO proposed	60	8.22%
	200	10.1%
	400	10.8%
